# Simulated online adaptive radiotherapy on C‐arm linacs: Integrating high resolution cone‐beam CT into abdominal stereotactic body radiation therapy

**DOI:** 10.1002/acm2.70615

**Published:** 2026-05-12

**Authors:** Shiqin Su, Song Gao, Xiao Liang, Hunter Mehrens, Kevin E. Casey, Zhiqian H. Yu, Yusung Kim, Comron J. Hassanzadeh, Chad Tang, Phuoc Tho Tran, Deborah Schofield, Peter Balter, Eun Young Han

**Affiliations:** ^1^ Department of Radiation Physics University of Texas MD Anderson Cancer Center Houston Texas USA; ^2^ Department of GU Radiation Oncology University of Texas MD Anderson Cancer Center Houston Texas USA

**Keywords:** adaptative radiotherapy, cone‐beam CT, hyperSight, rayStation

## Abstract

**Background:**

Conventional cone‐beam computed tomography (CBCT) systems, while providing adequate visualization for positioning, have historically been limited by image truncation, scatter contamination, image noise, geometric artifacts, and unreliable CT number accuracy, which preclude their routine use for accurate dose calculation and re‐planning. Recent advances in CBCT detector technology and image reconstruction algorithms have led to the development of next‐generation CBCT platforms with improved image quality and CT number stability.

**Purpose:**

This study aimed to develop and evaluate an efficient daily adaptation workflow on a standard C‐arm linear accelerator by integrating HyperSight CBCT and plan adaptation in RayStation.

**Methods:**

Image quality metrics and CT number accuracy were evaluated and compared with those of an institutional CT simulator and conventional CBCT. Dose calculation accuracy was validated through measurement. The HyperSight CBCT adaptive workflow used both deformable image registration and rigid image registration for contour propagation, followed by manual revision when necessary. The adapted plan was iteratively optimized in RayStation by using the fine‐tune module. End‐to‐end test was done with a spine phantom and a pelvis phantom. Retrospective evaluation of four abdominal SBRT cases (12 fractions) compared estimated delivered and adapted plans with respect to target coverage, organs at risk (OARs) sparing, and monitor unit (MU) usage.

**Results:**

Improved image quality and CT number accuracy compared to conventional CBCT were observed. The adaptation process required 13 ± 3 min per fraction from HyperSight‐CBCT acquisition to final plan evaluation, excluding time for contour revisions. Across all cases, adapted plans demonstrated improved dosimetric quality relative to delivered plans. Gross tumor volume V_100%_ and planning target volume V_100%_ were consistently higher in the adapted plans, with an average increase of 6.3% (*P *= 0.004). Doses to OARs, including the small bowel and ipsilateral kidney, were reduced or maintained. Adapted plan MU usage was comparable to reference plans, indicating no increase in treatment complexity.

**Conclusions:**

Integrating HyperSight CBCT with RayStation's streamlined plan adaptation capability provided dosimetric advantages. It improved target coverage and OAR sparing without increasing treatment complexity and represents a practical alternative to Ethos or MR‐Linac systems without the need for specialized delivery platforms.

## 1 INTRODUCTION

Adaptive radiation therapy represents a pivotal advancement in modern radiation oncology, addressing the substantial anatomic variations that can occur throughout the course of patient treatment.^1^, ^2^ These variations, ranging from tumor regression or progression to organ motion and patient weight change, can undermine the fidelity of the initial treatment plan, potentially compromising target coverage and increasing the dose to surrounding healthy tissues. Although onboard imaging, particularly cone‐beam computed tomography (CBCT), has key roles in image‐guided radiation therapy, conventional CBCT systems have been limited by truncation, poor image quality, low soft tissue contrast, and inaccurate CT number‐to‐relative electron density conversion.^3^ These deficiencies have restricted their use for precise dose calculation and limited the clinical implementation of adaptive planning on CBCT based systems.

The introduction of HyperSight (Varian Medical Systems, California) imaging technology represents a substantial technologic leap in onboard volumetric imaging. Initially integrated into Varian's Ethos and Halcyon platforms, HyperSight has demonstrated marked improvements in image clarity, acquisition speed, and consistency.^3^, ^4^ With recent FDA 510(k) clearance for use of HyperSight on TrueBeam and Edge linear accelerators (linacs), this advanced technology is now accessible to broader patient populations and clinical infrastructures.

The HyperSight package comprises both hardware and software advances, including a large 43 × 43 cm^2^ kV imager panel, increased gantry rotation speed (from 6°/s to 9°/s), iterative CBCT (iCBCT) reconstruction algorithms with scatter correction, metal artifact reduction (MAR), and extended field‐of‐view (FOV) reconstruction capabilities up to 70 cm in diameter. These improvements collectively enhance image quality, particularly for soft tissue visualization and anatomic delineation, which are critical for adaptive radiotherapy.

It is important to note that the installation of HyperSight on a C‐arm linear accelerator represents a fundamentally different system architecture and clinical workflow compared with dedicated online‐adaptive platforms. For instance, the Ethos system, designed specifically for online adaptive radiotherapy with integrated CBCT, has been extensively evaluated in the literatures, with numerous studies reporting on its imaging performance and clinical applications.^3^, ^4^, ^5^, ^6^, ^7^, ^8^, ^9^, ^10^, ^11^, ^12^ By contrast, HyperSight on a C‐arm linac is not inherently integrated with an online‐adaptive framework; however, when coupled with external adaptive planning tools, it has the great potential to support efficient and accurate plan adaptation. With the recent rollout of HyperSight on TrueBeam C‐arm linac systems, peer‐reviewed data characterizing its imaging performance, quantitative accuracy, and clinical implications in this setting remain limited. Given the widespread adoption of C‐arm linacs globally, compared to Ethos or MR‐linacs, a comprehensive evaluation of HyperSight‐enabled adaptive workflows in this setting is both timely and necessary.

This study aimed to address the current gap by systematically evaluating the imaging performance of HyperSight CBCT on a TrueBeam linac and by developing a clinical workflow integrating HyperSight CBCT with adaptive plan generation using the RayStation treatment planning system (v12B, RaySearch Laboratories, Sweden). The goal was to establish a practical and efficient adaptive process that maintains high dosimetric quality while minimizing plan‐generation time. Through combined image‐quality assessment, workflow development, and retrospective plan comparison, this work lays the foundation for broader clinical implementation of HyperSight‐guided adaptive radiotherapy on standard C‐arm linac platforms.

## 2 METHODS AND MATERIALS

### 2.1 Hypersight CBCT image performance

A Varian TrueBeam linac was upgraded from version 2.7 MR4 to version 4.1, and the HyperSight imaging system was installed in this unit. The upgrade included software, the gantry rotation motor, and the kVp imaging panel. Specifications for HyperSight and the previous‐generation CBCT on a TrueBeam (v2.7) are compared in Table 1. HyperSight incorporates advanced iterative reconstruction with scatter correction and MAR algorithm (Figure S1). A comprehensive evaluation was conducted to assess the image quality of the HyperSight imaging system, focusing on CT number accuracy, geometric distortion, image contrast, spatial resolution, uniformity, and noise reduction. Image quality except CT number accuracy was assessed by using a CT ACR 464 phantom​ (Sun Nuclear Corporation, Florida) with iCBCT and MAR algorithms and different combinations of noise suppression and smoothing, which are unique to HyperSight. This allowed flexibility in determining the optimal reconstruction configurations to be determined for specific clinical scenarios, particularly those in which soft tissue contrast is crucial. The longitudinal performance of HyperSight iCBCT was also compared against a CT simulator (Philips Brilliance Big Bore CT simulator, Philips Healthcare, Netherlands) and a previous‐generation CBCT system on a TrueBeam (v2.7) by using Catphan 604 (The Phantom Laboratory, New York). All image analyses were done in SunCheck (Sun Nuclear Corporation, Florida).

**TABLE 1:** Comparison of specifications for HyperSight and previous‐generation CBCT on a TrueBeam system.

CBCT hardware	HyperSight	Previous Generation
Active imaging area, cm^2^	43×43	40×30
Pixel matrix	3072×3072	1424×1072
Gantry rotation speed, deg/s	9	6
Scan duration, s	16.6 – 40	24 ‐ 60
Reconstruction algorithm	FDK, iCBCT, ICBCT MAR	FDK, iCBCT
Reconstruction FOV diameter, cm	27 (head); 48 (body)	25 (head); 46 (body)
Reconstruction length, cm	24.5 (head); 26 (body)	17 (head); 18 (body)
Extended FOV, cm	Up to 70	N/A

Abbreviations: CBCT, cone‐beam computed tomography; FDK, Feldkamp‐Davis‐Kress; FOV, field of view; iCBCT, iterative CBCT; MAR, metal artifact reduction.

### 2.2 CT number and dose calculation accuracy

The accuracy of HyperSight's CT number ‐to‐relative electron density conversion was evaluated by using a tissue characterization phantom (Gammex 467, Sun Nuclear Corporation, Florida), which includes inserts composed of tissue‐equivalent materials with known electron density densities relative to water. HyperSight iCBCT images were acquired across three imaging reconstruction methods: HyperSight‐iCBCT, HyperSight‐iCBCT with MAR, and conventional HyperSight‐FDK at 125 kV (pelvis) and 140 kV (large pelvis). Five circular regions of interest (radius 11 mm) were placed in the uniform module for noise analysis (defined as the standard deviation of CT number). CT number (average region of interest) of each insert were measured and compared against reference CT number derived from the institution's CT simulator calibration curve, acquired at 120 and 140 kV. Deviations in CT numbers between HyperSight iCBCT and the CT simulator were evaluated across multiple reconstruction methods. Dose calculations based on HyperSight CBCT were compared with those from the CT simulator, with doses measured using ionization chambers.

### 2.3 Novel adaptation workflow on C‐arm linac

**FIGURE 1:**
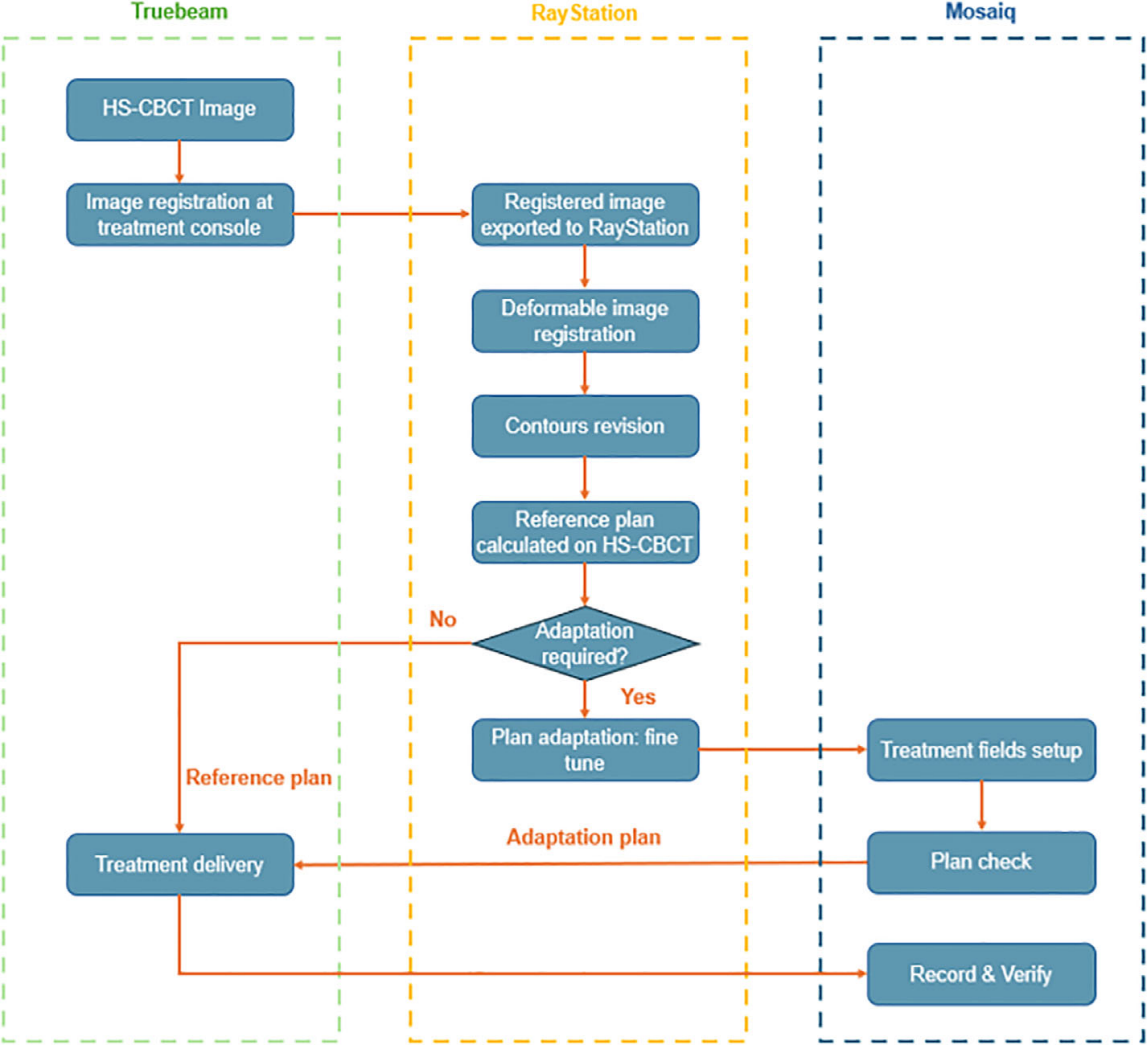
The adaptive treatment workflow using HyperSight. Contours are transferred to daily image through rigid or deformable image registration. The adapted plan is then adapted iteratively using RayStation's “fine‐tuned” optimization. The plan is then exported to Mobius for intensity‐modulated radiation therapy (IMRT) quality assurance (QA), to MOSAIQ for recording, and to TrueBeam for delivery.

The C‐arm linac adaptation workflow is illustrated in Figure 1. The CBCT image was acquired at the imaging isocenter, after which the treatment couch was shifted to the planned treatment isocenter based on image registration performed at the treatment console. This resulting treatment isocenter was then used as the reference isocenter for adaptive dose recalculation and replanning. The CBCT images, together with the corresponding isocenter information, were imported into the RayStation TPS via rigid registration. The target volumes and supporting couch structures were copied through the rigid registration, and contours for organs at risk (OARs) were deformed by using RayStation's deformable image registration algorithm to reflect the daily anatomy captured in the HyperSight iCBCT. The physician reviewed and modified targets and OARs contours if necessary. The initial planned dose was first recalculated on the daily HyperSight iCBCT image to assess delivered dose and determine whether plan adaptation was required. If the recalculated dose demonstrated significant deviation from clinical objectives (e.g., compromised target coverage or excessive OAR dose), the adaptive planning process was initiated using RayStation's Fine Tune Optimization module.^13^ This optimization tool is designed to enhance treatment plan quality by selectively improving underperforming clinical goals while using the dose distribution of the original plan as a voxel‐wise reference. This ensures that the adapted dose distribution remains clinically consistent with the intended treatment while accommodating daily anatomical changes. The approved plan was manually exported to the MOSAIQ Record and Verify system (Elekta, Sweden) for setup and delivery. Patient‐specific IMRT quality assurance (QA) using Mobius3D (Varian Medical Systems, California) and physics plan check were also performed to ensure the plan quality.

The proposed adaptation workflow included a two‐step commissioning process. First, a customized spine phantom (RTsafe, Greece) and a pelvis phantom (Brainlab Medical Solutions, Germany) were used to assess the basic functionality of HyperSight CBCT in a controlled environment. This included end‐to‐end tests to evaluate consistency and dose accuracy, including data transfer, patient‐specific IMRT QA and dose delivery. Second, retrospective clinical evaluation was performed by comparing HyperSight‐based to CT simulator‐based adaptation (Figure S1) and using four abdominal SBRT patients over a total of 12 fractions. Adaptation was demanded with the following criteria: gross tumor volume (GTV) V100% < 95%, small bowel D0.03cm^3^ > 32 Gy, or ipsilateral kidney‐GTV V10Gy > 40%. Wilcoxon signed‐rank tests were performed to assess the statistical significance of changes to tumor coverage. Dose‐volume metrics for targets (e.g., planning target volume [PTV], GTV) and critical OARs (e.g., bowel, kidney) were analyzed to evaluate the clinical effects of adaptation. Monitor unit (MU) usage was also compared between the original and adapted plans to assess any increase in plan complexity resulting from adaptation.

## 3 RESULTS

### 3.1 HyperSight CBCT image performance

Table 2 summarizes the quantitative parameters obtained from image quality measurements performed on the Gammex Model 464 phantom for various combinations of iCBCT reconstruction methods, including filters (standard, smooth, and sharp), noise suppression (medium, high, and very high), and MAR. A conventional Feldkamp‐Davis‐Kress (FDK) reconstruction on HyperSight CBCT was also presented as a reference.

**TABLE 2:** Image quality of various reconstruction modes using HyperSight CBCT on an ACR 464 phantom.

		HyperSight‐iCBCT				HyperSight‐iCBCT MAR		
	Filter	Standard	Smooth	Smooth	Sharp	Smooth	Smooth	
	Noise suppression	Medium	Medium	High	Very high	Medium	High	HyperSight‐FDK
	Geometric distortion, mm	0.44	0.13	0.13	0.03	0.13	0.13	0.54
Pelvis	Spatial resolution, lp/mm	0.46	0.46	0.45	0.46	0.45	0.44	0.50
125 kV	Uniformity, HU	−2.3	−1.4	−5.2	1.7	−1.8	−1.6	−10.1
1060.8 mAS	Noise, HU	4.27	2.19	1.40	4.00	1.88	1.46	5.10
	Low contrast (0.6%), mm	>6.0	4.5	4.5	5.0	4.5	4.5	> 6.0
	Geometric distortion, mm	0.80	0.44	0.44	0.40	0.40	0.40	0.60
Pelvis Large	Spatial resolution, lp/mm	0.47	0.50	0.50	0.50	0.50	0.50	0.50
140 kV	Uniformity, HU	−5.6	−8.7	−8.4	−5.8	−5.2	−4.5	−10.2
1657.5 mAS	Noise, HU	2.98	1.80	1.60	3.30	1.80	1.60	4.80
	Low contrast (0.6%), mm	5.5	4.0	4.0	4.0	5.0	4.0	>6.0

Abbreviations: FDK, Feldkamp‐Davis‐Kress reconstruction; HU, Hounsfield units; iCBCT, iterative cone‐beam computed tomography; lp, line pairs; MAR, metal artifact reduction.

All imaging protocols demonstrated submillimeter geometric accuracy. For the pelvis protocol (at 125 kV), the measured geometric distortion ranged from 0.03 to 0.44 mm (iCBCT) compared to 0.54 mm (FDK reconstruction), indicating excellent geometric accuracy across reconstruction settings. Spatial resolution, expressed in line pairs per millimeter (lp/mm), was consistent across iCBCT modes, with conventional reconstruction achieving slightly higher resolution (0.50 lp/mm), demonstrating minimal impact from filter or MAR utilization. Uniformity values, reported as the mean CT number difference between the phantom center and periphery, ranged from –5.2 HU to 1.7 HU among the iCBCT reconstructions, whereas the conventional reconstruction showed a larger deviation (–10.1 HU). The noise suppression algorithm effectively reduced the noise level when combined with the smooth filter. However, this was not the case with the sharp filter, which reduced geometric distortion at the cost of higher noise. Similar results were observed for the large pelvis protocol acquired at 140 kV.

The performance of HyperSight‐iCBCT was monitored monthly from November 2024 through May 2025 using a Catphan phantom and compared to results from a CT simulator and previous‐generation CBCT (Figure S2). Across the 7‐month period, HyperSight iCBCT demonstrated excellent stability, with all image quality metrics remaining within tight tolerance bands relative to the baseline.

### 3.2 CT number and dose calculation accuracy

**FIGURE 2:**
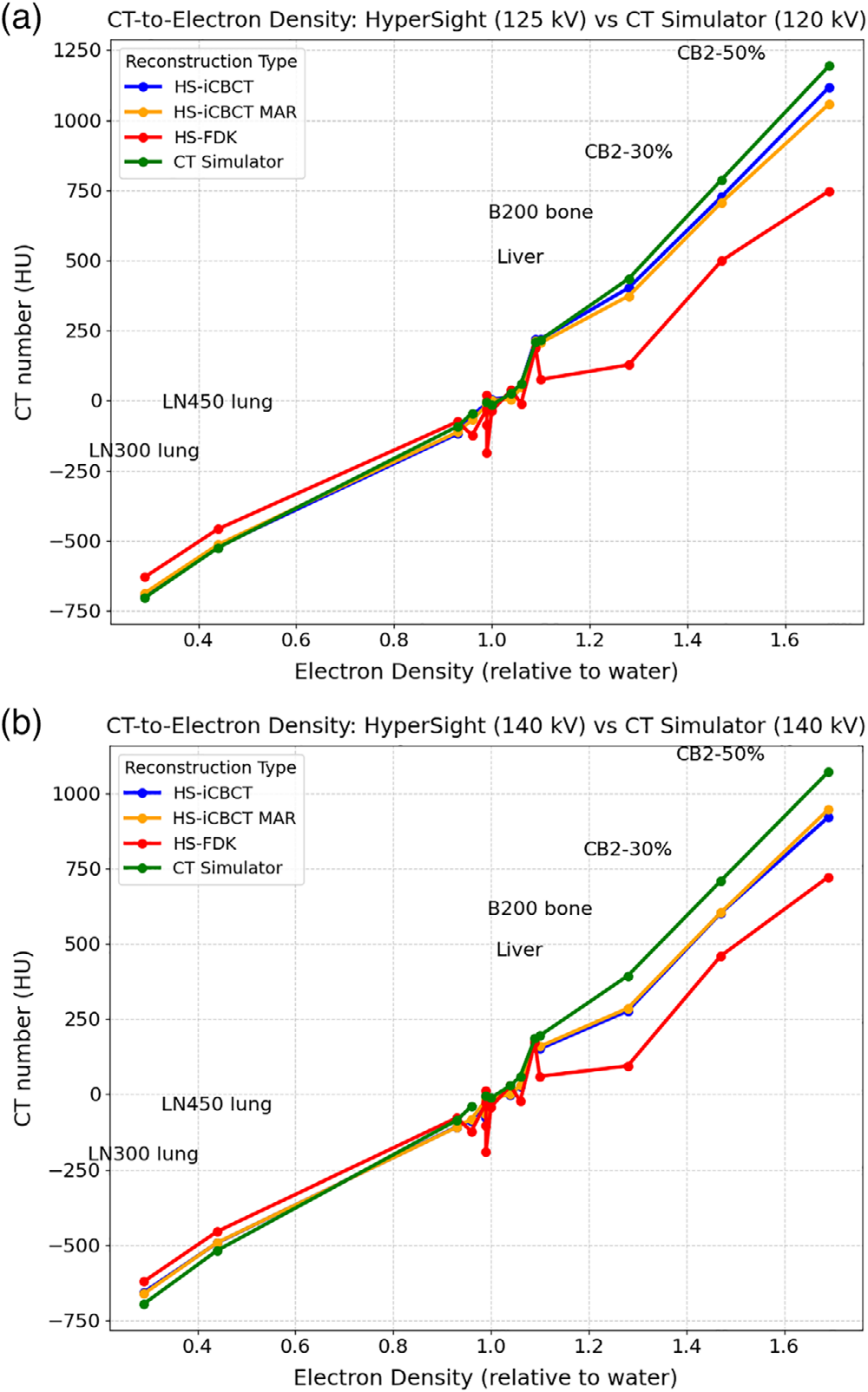
CT number‐to‐relative electron density conversion for HyperSight (HS) ‐iCBCT, HS‐iCBCT metal artifact reduction (MAR), and HS‐ Feldkamp‐Davis‐Kress reconstruction (FDK) reconstruction acquired (top): at 125 kV, compared to a CT simulator at 120 kV and (bottom): at 140 kV, compared to a CT simulator at 140 kV.

Across all materials, both HyperSight‐iCBCT and HyperSight‐iCBCT MAR reconstruction exhibited markedly improved CT number linearity and accuracy compared with the HyperSight‐FDK reconstruction (Figure 2). For the pelvis protocol, the mean absolute CT number deviation from the CT simulator reference was 26.7 HU for HyperSight‐iCBCT, 32.8 HU for HyperSight‐iCBCT MAR, and 92.2 HU for HyperSight‐FDK, indicating about 70% reduction in HU error for HyperSight‐iCBCT relative to HyperSight‐FDK. For soft‐tissue materials (water, liver, breast, and solid water), HyperSight‐iCBCT achieved CT number within ± 20.0 HU of the reference CT, whereas HyperSight‐FDK exhibited deviations exceeding 80.0 HU in several inserts. For lung‐equivalent materials (LN300 and LN450), CT number differences relative to the reference CT were within 10.0 HU for both HyperSight‐iCBCT modes but exceeded 60.0 HU for HyperSight‐FDK. For bone‐equivalent materials (B200, CB2‐30%, CB2‐50%), HyperSight‐iCBCT demonstrated reduced CT number bias. The high‐density bone material (CB2‐50%) showed an CT number difference of –60.7 HU for HyperSight‐iCBCT compared with –288.5 HU for HyperSight‐FDK, representing a 79% improvement in CT number accuracy. Similarly, the mid‐density bone material (CB2‐30%) showed improvement from –307.0 HU (HyperSight‐FDK) to –33.0 HU (HyperSight‐iCBCT). The inclusion of MAR slightly reduced metal‐related streak artifacts but did not affect CT number accuracy compared with the HyperSight‐iCBCT non‐MAR reconstruction. CT number of selected materials was also compared among different reconstructions, and no significant CT number discrepancies were measured (Supplementary Table S3).

The improved CT number accuracy of iCBCT translates into a near linear relationship between CT number and relative electron density. The CT number‐to‐relative electron density conversion from iCBCT closely followed that of the reference CT, particularly in the clinically relevant density range (0.30–1.20 g/cm^3^). HyperSight‐FDK, in contrast, showed substantial CT number deviation, especially at higher densities, leading to reduced dosimetric accuracy if used for treatment planning or dose calculation.

**FIGURE 3:**
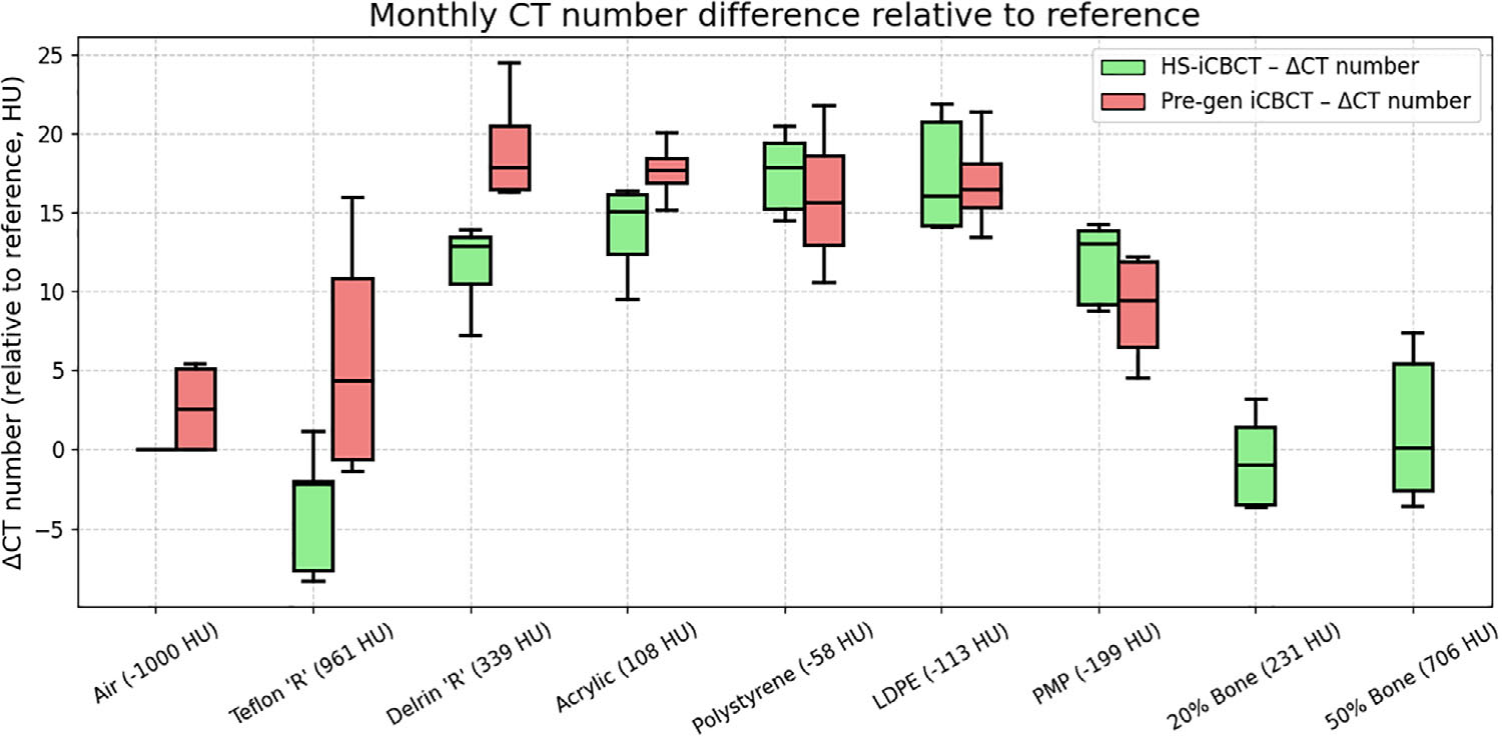
Longitudinal (long‐term) CT number deviation of HyperSight iCBCT on a CatPhan against CT simulator baseline, with iCBCT on previous generation CBCT as reference. Note that 20% bone and 50% bone were not measured in routine monthly QA for the iCBCT on previous generation CBCT. iCBCT, iterative cone‐beam CT. LDPE, low‐density polyethylene; PMP, polymethylpentene.

Longitudinal CT number deviation against the institution baseline for several tissue‐equivalent materials was analyzed and were compared with iCBCT on previous‐generation CBCT (Figure 3). Across all inserts, HyperSight‐iCBCT CT number remained stable with no observable drift over the 7‐month observation period. The mean absolute CT number deviation from baseline was 8.4 HU. HyperSight CT numbers ranged from 955–965 HU for Teflon and 350–360 HU for Delrin, both high‐density materials, differing by < 3% from the CT simulator. This represents a notable improvement compared with previous generation iCBCT. Deviations for soft‐tissue equivalent materials (acrylic, polystyrene) and low‐density materials (low‐density polyethylene [LDPE], polymethylpentene [PMP]) were within ± 20 HU and were comparable to previous‐generation iCBCT.

#### 3.2.1 Spine phantom study

**FIGURE 4:**
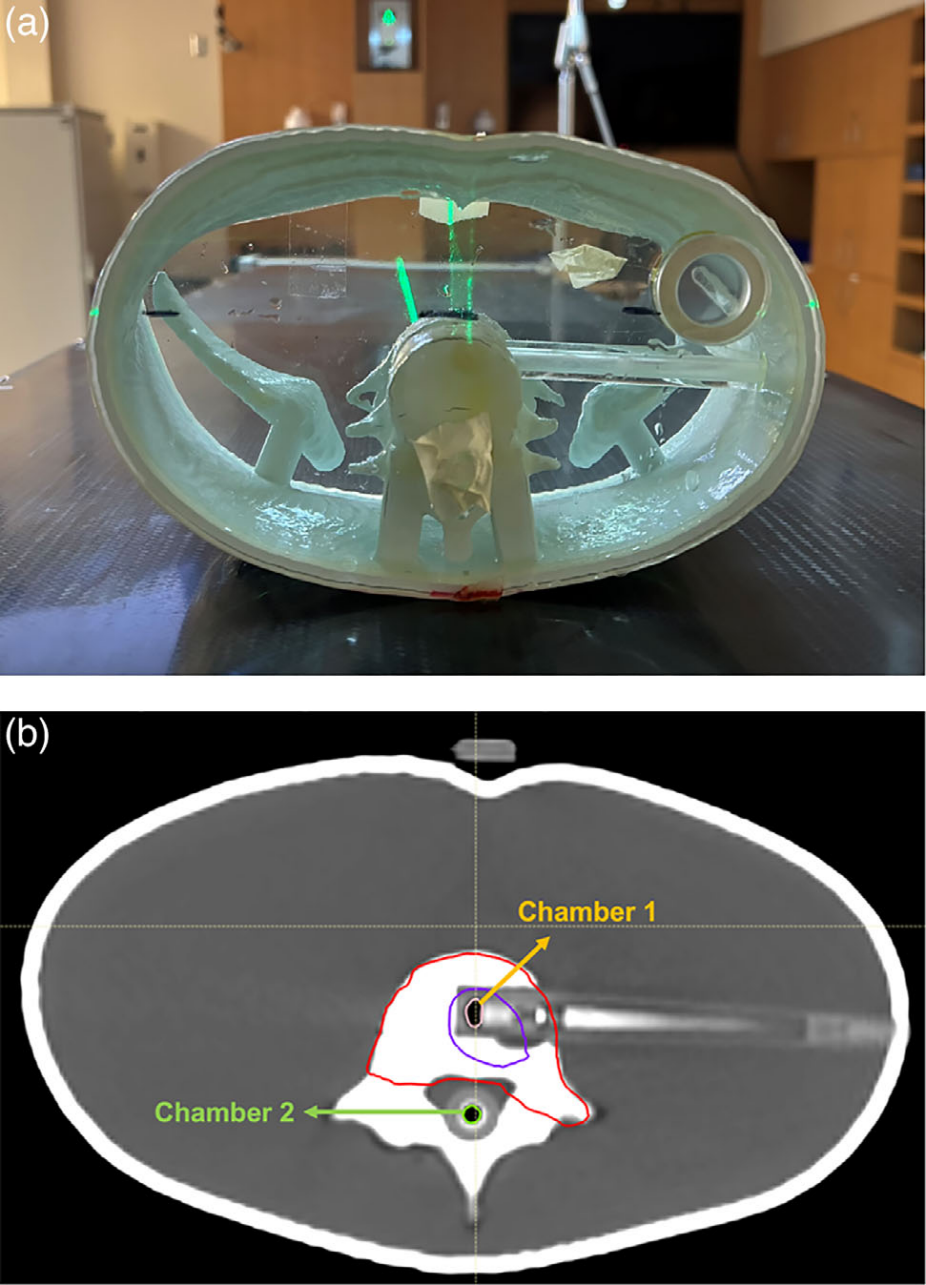
Spine phantom for heterogeneity correction test, with two ionization chamber insertions.

The accuracy of dose calculation was further evaluated on an spine phantom, with two ion chambers inserted within the target (high‐dose region) and at the spinal cord (high‐gradient region), separately (Figure 4). The target dose was within 1.9% of expected (measurement 1320.86 cGy vs calculation 1295.50 cGy) while the cord dose was within 1.7% of expected (measurement 356.51 cGy vs. calculation 350.50 cGy). Gamma analysis of plans on HS‐iCBCT images and CT images showed 100% pass rate for 1%,1 mm, and 10% global threshold^1^ A similar test was also performed on a pelvis phantom present in Figure S4.

### 3.3 Novel adaptation workflow on C‐arm linac

The complete adaptation process took 13 ± 3 min for the 12 test fractions, including DICOM export/import, deformable image registration, plan recalculation/optimization, plan review. CBCT alignment and contour modification were excluded from time study, as they vary on a case‐by‐case basis.

A comparison of clinically delivered and adaptive plans was summarized in Table 3, four abdominal stereotactic body radiation therapy cases (fraction 3–5 of a patient with 50 Gy in 5 fractions, 2 patients with 42 Gy in 3 fractions, and 1 patient with 36 Gy in 3 fractions). Across all four cases, adaptive planning led to improved dosimetric outcomes compared with delivered plans. The adapted plans consistently increased target coverage, with the GTV V_100%_ and PTV V_100%_ values either matching or exceeding those of the delivered plans, with an average increase of 6.3% for GTV V_100%_. Wilcoxon signed‐rank tests showed the statistical significance of the improvement of GTV V_100%_ with adaptation (*P *= 0.004). IMRT QA using Mobius3D showed a 99.7 ± 0.2% gamma pass rate (3%/2 mm/10 % global threshold).

**TABLE 3:** Comparison of delivered plans (reference plan recalculated on daily HyperSight‐CBCT), adapted plans, and the reference plans for targets and organs at risk.

		GTV V_100%_ (%)	PTV V_100%_ (%)	Small bowel D_0.03cm3_ (Gy)	Ipsi Kidney‐GTVV_10Gy_ (%)	MUs
	Reference	90.01	90.25	28.95	35.72	5013
Case 1	Delivered	93.4 (90.0‐93.7)	92.3 (90.0‐92.5)	30.0 (28.4‐32.5)	38.2 (35.1‐39.1)	5013
	Adapted	94.3 (94.0‐95.0)	92.0 (90.0‐93.5)	30.0 (23.4, 31.9)	35.5 (34.9‐36.1)	4964 (4781‐5013)
	Reference	100	95.18		39.15	4480
Case 2	Delivered	100 (100‐100)	97.6 (95.7‐98.5)		40.8 (38.0‐42.6)	4480
	Adapted	100 (100‐100)	97.7 (95.7‐98.4)		38.0 (36.5‐38.6)	4387 (4385‐4480)
	Reference	90.05	92.2	29.84	10.35	2494
Case 3	Delivered	84.0 (80.0‐89.0)	84.6 (84.1‐90.2)	35.0 (30.4‐36.2)	11.7 (10.2‐14.9)	2494
	Adapted	91.2 (92.1‐93.1)	91.8 (90.1‐94.4)	28.8 (28.8‐29.2)	12.6 (12.3‐13.7)	2944 (2431‐2603)
	Reference	98.81	98.99	19.08	32.08	2678
Case 4	Delivered	99.9 (72.4‐100.0)	99.6 (74.6‐99.8)	19.4 (19.2‐19.5)	37.9 (32.8‐40.8)	2678
	Adapted	99.9 (93.0‐100.0)	99.6 (96.3‐99.8)	20.0 (12.9‐21.0)	35.3 (31.5‐37.9)	2678 (2597‐2692)

Abbreviations: GTV, gross tumor volume; Ipsi, ipsilateral; PTV, planning target volume.

## 4 DISCUSSION

The introduction of HyperSight CBCT onto the TrueBeam platform has enhanced imaging workflow and hardware capabilities that support efficient and accurate adaptive radiation therapy on a traditional C‐arm linac. One of the major goals of this upgrade was to enable high‐quality CBCT imaging with sufficient soft tissue contrast and CT number accuracy to support direct dose calculation.

In our early clinical experience, the image quality of HyperSight CBCT with standard iCBCT reconstruction (on TrueBeam v4.1) was largely comparable to that of iCBCT on the previous‐generation CBCT (on TrueBeam v2.7), while both filter settings and noise suppression unique to HyperSight system reduced geometric distortion compared with the standard iCBCT reconstruction. In addition to those improvements, HyperSight introduces a larger field of view (up to 70 cm in diameter), shorter acquisition time (16.6 and 40 sec for half and full rotation, respectively), and MAR, collectively enabling more consistent and efficient image acquisition across diverse clinical scenarios. However, the improvements were less pronounced compared to the HyperSight on an Ethos‐based machine.^4^, ^5^ For extended FOV reconstruction beyond the physical reconstruction diameter (48.5 – 70 cm), geometric accuracy can be reduced,^14^ therefore geometric uncertainties should be understood for clinical use. Compared to MR‐linac systems, which offer superior soft‐tissue contrast, HyperSight provides a larger effective field of view, shorter acquisition times, and robust MAR, enabling rapid volumetric imaging across a wide range of treatment sites. In addition, our workflow can be selectively applied based on clinical need, whereas MR‐linac users must choose from adapt‐to‐position or adapt‐to‐shape workflows.

CT number accuracy has also improved with the use of HyperSight iCBCT on the TrueBeam and was consistent with findings reported for HyperSight installed on the Ethos and Halcyon systems.^15^, ^16^, ^17^ Our evaluations show that the CT numbers obtained from iCBCT scans are closer to those from diagnostic‐quality CT simulators compared to FDK reconstruction or iCBCT on previous generation CBCT. We also observed that variations in reconstruction settings had minimal effects on CT numbers, which allows optimization of visual appearance (e.g., enhancing soft tissue contrast or reducing noise) without compromising dosimetric consistency. Despite these improvements, CT number accuracy of HyperSight iCBCT on a C‐arm linac remains inferior to that of a CT simulator in high‐density regions and in the vicinity of implants. This limitation should be considered when using CBCT for direct dose calculation in high‐precision treatments or when significant density heterogeneities are present.

With regard to the adaptation workflow, the integration of HyperSight imaging capabilities to our TrueBeam system has introduced meaningful advancements in image‐guided radiotherapy, particularly in the context of adaptive radiation therapy. With enhanced CT number accuracy, expanded field of view, and improved soft tissue contrast of HyperSight‐iCBCT, the feasibility of accurate dose recalculation and adaptation using the patient's daily anatomy has improved. When combined with RayStation's Fine‐Tune Optimization and deformable image registration tools, a practical workflow for offline or online adaptive planning becomes available on a conventional C‐arm linac. In the retrospective study, doses to OARs, such as the small bowel and ipsilateral kidney, were reduced or maintained, demonstrating improved sparing. The MUs remained comparable with those of the reference plans, indicating that these benefits were achieved without increased treatment complexity.

One key advantage of this approach is its flexibility and modularity. Unlike integrated platforms such as Ethos, which have a fixed workflow and limited customization, RayStation enables tailored planning strategies, user‐defined contour adaptation, and multi‐criteria optimization schemes. The fine‐tune optimization specifically enables selective plan improvement, optimizing only unmet clinical goals while preserving dosimetrically acceptable structures, which is crucial for maintaining consistency across fractions and for avoiding unnecessary full re‐optimization. This flexibility is particularly advantageous in timely and complex clinical scenarios in which patient‐specific factors require bespoke planning solutions.

This approach can also leverage existing infrastructure, avoiding the need to invest in a new delivery platform. Many clinics already equipped with TrueBeam systems can potentially adopt online adaptive workflows incrementally, using familiar software and hardware components. RayStation's scripting capabilities and dose tracking tools further enhance the ability to implement adaptive protocols in a semi‐automated fashion.

However, several limitations must be acknowledged. First, workflow integration remains fragmented. Unlike Ethos, which offers a seamless “scan‐plan‐treat” pipeline within a single user interface and hardware ecosystem, the TrueBeam–RayStation workflow involves numerous systems (CBCT acquisition, image transfer, registration, planning, and QA), which can introduce inefficiencies (< 20 min from CBCT taken to adaptation plan exported, excluding contour modification by physicians) and increase the potential for error. This fragmented process may extend adaptation turnaround time, making online adaptive radiation therapy challenging without workflow automation. QA complexity also increases in non‐integrated workflows. Although the Ethos systems include embedded QA mechanisms, workflows using a TrueBeam and RayStation combination require independent validation steps to ensure that the adapted plans are safe and deliverable, including secondary dose verification and plan checks outside the treatment suite.

Second, at present, there is no consensus regarding the optimal criteria for triggering adaptation, and decisions are largely driven by physician judgment on a case‐by‐case basis. This variability reflects the necessity of broadly accepted clinical guidelines or quantitative thresholds defining when anatomical changes translate into clinically meaningful dosimetric impact.^18^, ^19^, ^20^ As a result, while adaptation‐on‐demand can be highly responsive to patient‐specific needs, its implementation remains heterogeneous across institutions and dependent on local expertise and workflow design.

Finally, the RayStation–based workflow also introduces important operational challenges and requires manual review and decision‐making at several steps, including real‐time clinical judgment to determine when anatomical changes are dosimetrically significant, deformable image registration accuracy, contour editing, and plan approval. These decisions are often made at the treatment machine under time pressure, potentially increasing cognitive load for therapists, dosimetrists, physicists, and physicians. Without clearly defined decision thresholds and standardized response pathways, variability in practice may arise across providers and treatment sites. This requirement has driven future work on improving workflow efficiency and introducing clinical decision points for implementing adaptive therapy.

## 5 CONCLUSIONS

This proof‐of‐concept study demonstrates that combining HyperSight CBCT with RayStation enables adaptive radiation therapy on C‐arm linacs, offering a flexible alternative to dedicated adaptive platforms such as Ethos or MR‐Linac. HyperSight on a TrueBeam linac enables the acquisition of CBCT images with improved soft tissue contrast and CT number accuracy, allowing direct dose calculation on the CBCT images. The workflow allowed timely adaptation of both targets and OARs, improving target coverage while reducing dose to critical structures. Although current limitations in software integration and automation remain, these findings highlight clinical feasibility and potential for broader adoption of adaptive therapy on conventional linac platforms.

## AUTHOR CONTRIBUTIONS

S.S. and E.H. contributed to the design of the study; C.H, C.T., and PP.T. contributed to patient recruitment and image review; S.G., D.S., H.M., and P.B., contributed to data acquisition: S.S., K.C., X.L., and E.H. contributed to data processing, and S.S., H.Y., Y.K., and E.H. contributed to the analysis and interpretation of data. S.S. drafted the manuscript and all authors critically reviewed and revised it. All authors approved the final version to be published and agree to be accountable for all aspects of the work in ensuring that questions related to the accuracy or integrity of any part of the work are appropriately investigated and resolved.

## CONFLICT OF INTEREST STATEMENT

Conflict of interest has been disclosed in the “Detailed Information” page during submission.

## Supporting information



Supporting Information

Supporting Information

## Footnotes

1 Note that this criterion was intentionally selected to probe worst‐case sensitivity and local dose differences, and differs from the institution's standard clinical IMRT QA criterion of 3%/2 mm with a 10% global threshold.
