# Supplementary figures and images for: Simulated online adaptive radiotherapy on C‐arm linacs: Integrating high resolution cone‐beam CT into abdominal stereotactic body radiation therapy
Source: J Appl Clin Med Phys. 2026 May 12;27(5):e70615. doi: 10.1002/acm2.70615 (PMC13167249; doi:10.1002/acm2.70615)

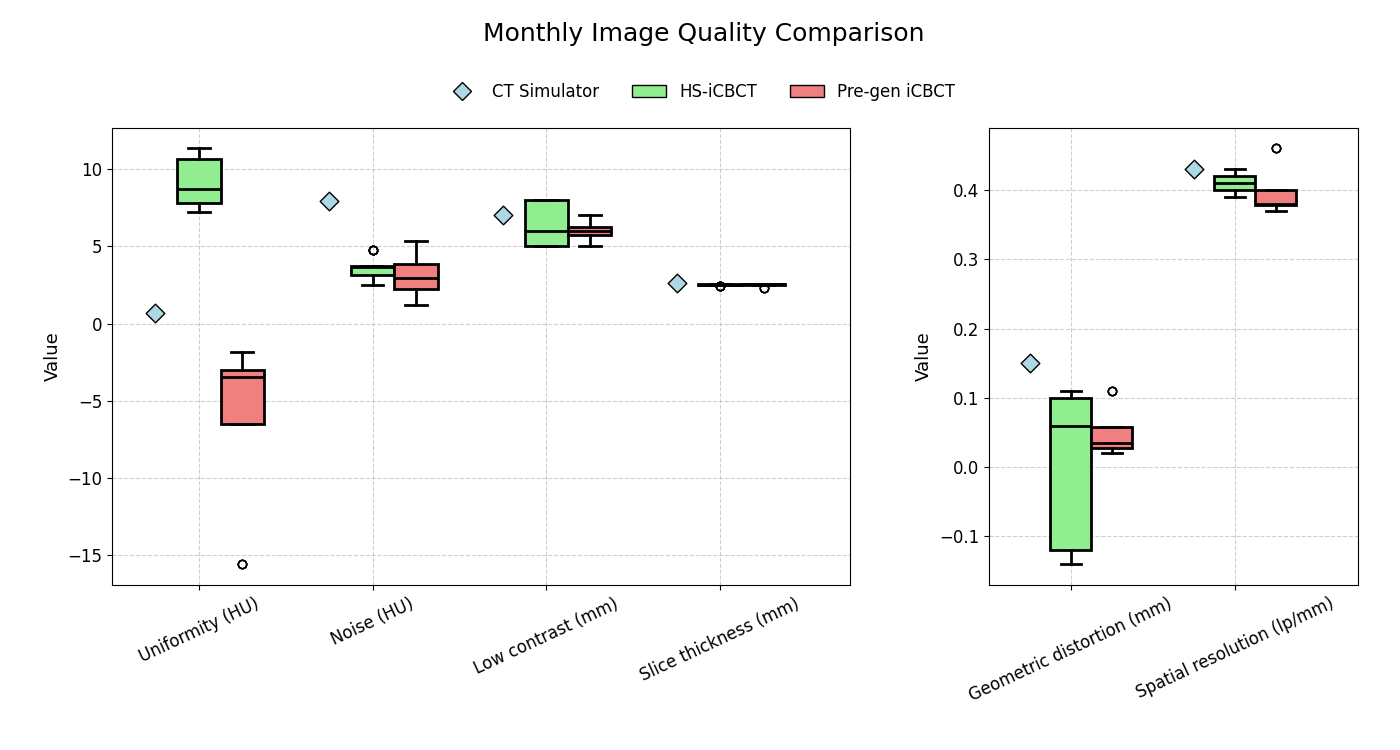

Supplement: Supplementary file 1 — Supporting Information [file ACM2-27-e70615-s002.zip › acm270615-sup-0002-Figure-S02.png]

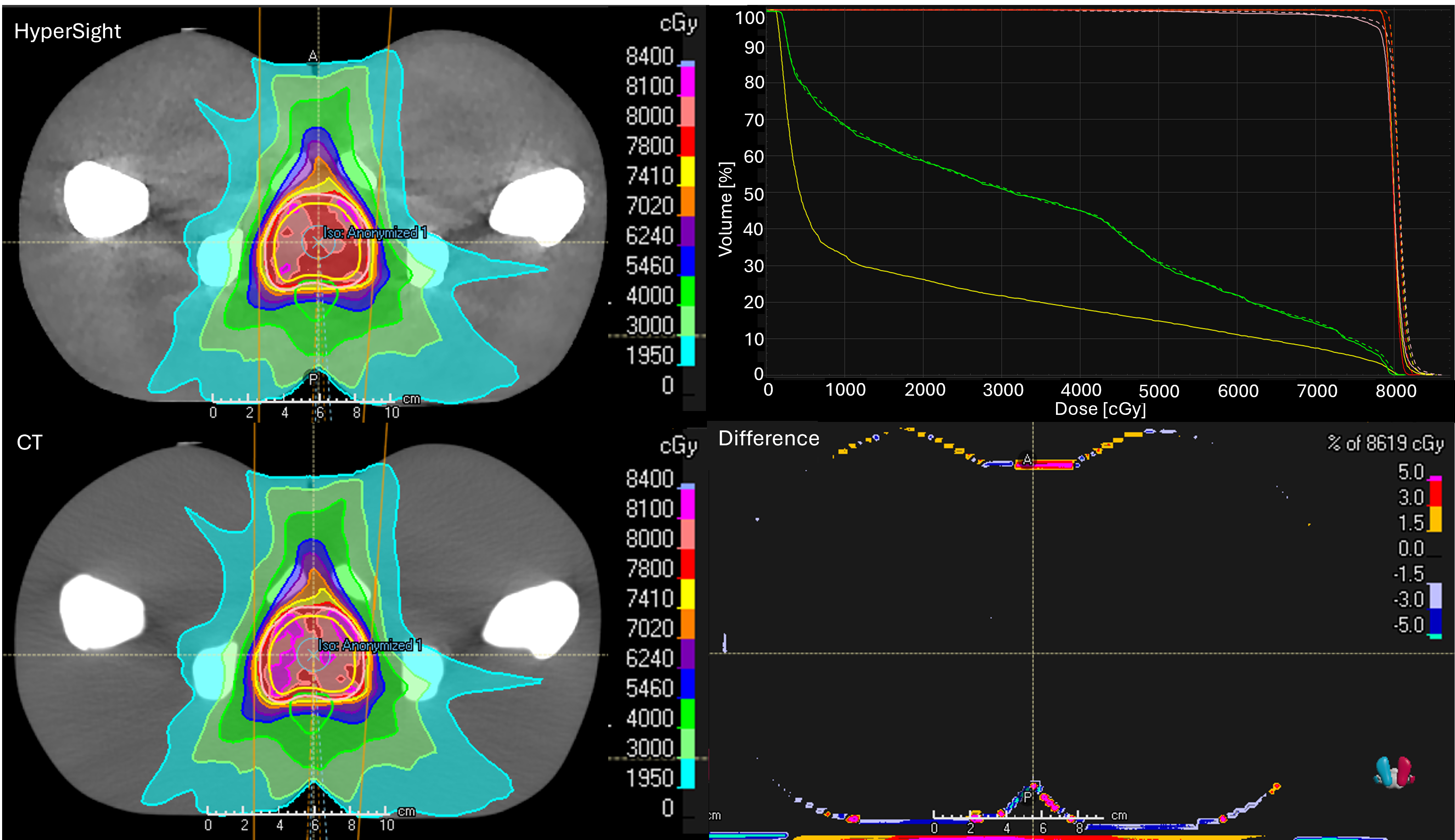

Supplement: Supplementary file 1 — Supporting Information [file ACM2-27-e70615-s002.zip › acm270615-sup-0003-Figure-S03.png]

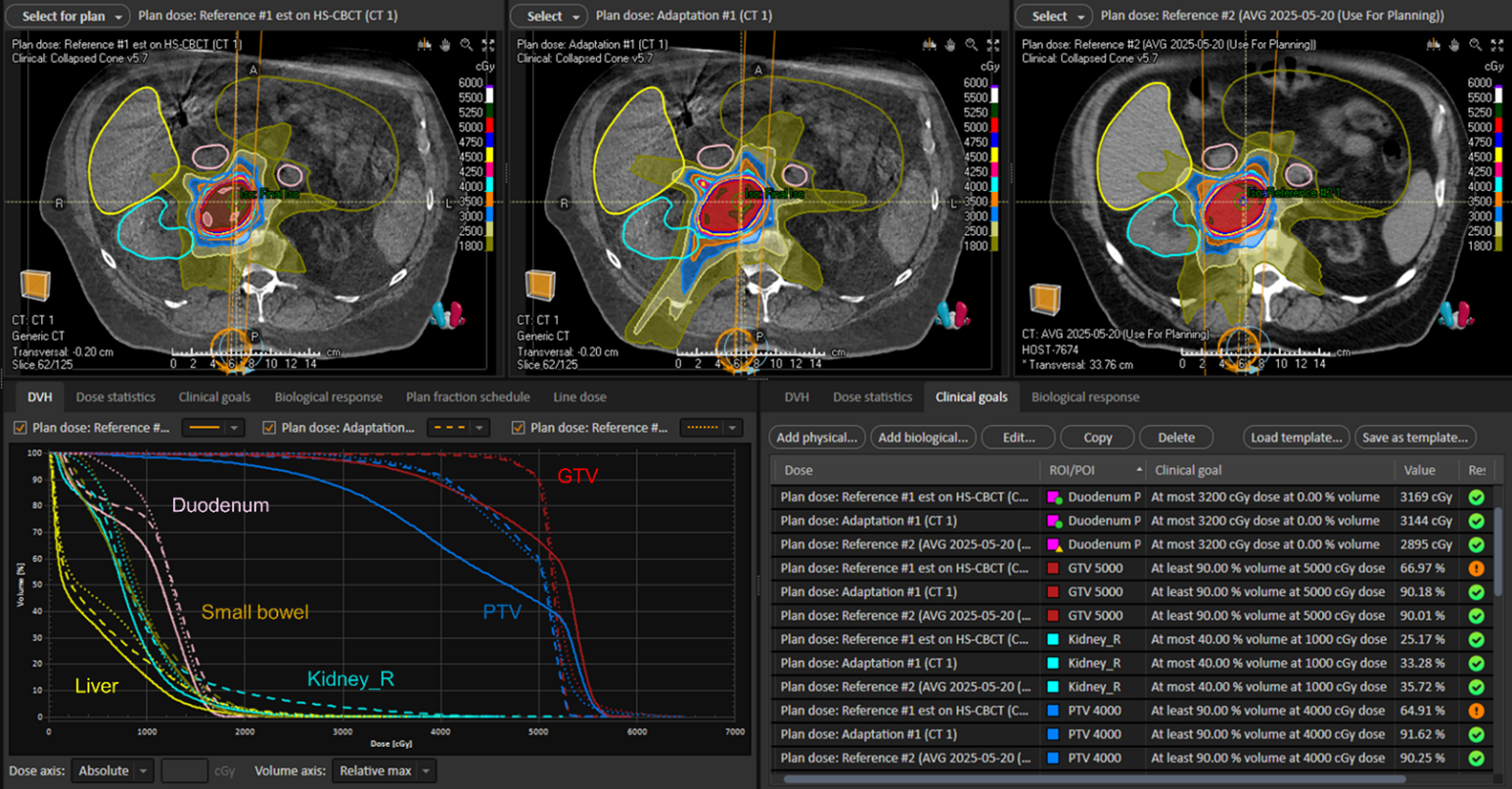

Supplement: Supplementary file 1 — Supporting Information [file ACM2-27-e70615-s002.zip › acm270615-sup-0004-Figure-S04.png]

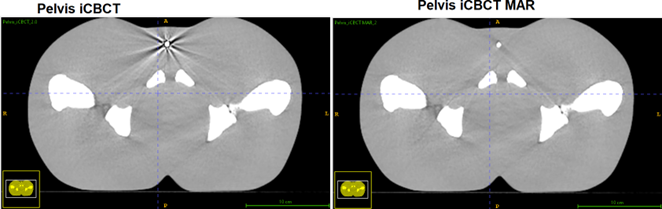

Supplement: Supplementary file 1 — Supporting Information [file ACM2-27-e70615-s002.zip › acm270615-sup-0001-Figure-S01.png]
