# Supplementary material for: Simulated online adaptive radiotherapy on C‐arm linacs: Integrating high resolution cone‐beam CT into abdominal stereotactic body radiation therapy
Source: J Appl Clin Med Phys. 2026 May 12;27(5):e70615. doi: 10.1002/acm2.70615 (PMC13167249; doi:10.1002/acm2.70615)
Supplement: Supplementary file 2 — Supporting Information [file ACM2-27-e70615-s001.docx]

Supplementary Table S3. HU values of various reconstruction mode using HyperSight on an ACR 464 Phantom

|  |  | **HS-iCBCT** | | | | **HS-iCBCT MAR** | | **HS-FDK** |
| --- | --- | --- | --- | --- | --- | --- | --- | --- |
|  | Filter | Standard | Smooth | Smooth | Sharp | Smooth | Smooth |  |
|  | Noise Suppression | Medium | Medium | High | Very high | Medium | High |  |
|  | Air, HU | –994.89 | –997.35 | –997.6 | –999.79 | –996.91 | –997.21 | –988.5 |
| **Pelvis** | Acrylic, HU | 120.28 | 120.24 | 121.8 | 119.7 | 121.87 | 122.12 | 123.8 |
| **125 kV** | Bone, HU | 958.9 | 956.57 | 951 | 962.61 | 961.41 | 961.34 | 948.7 |
| **mAS 1060.8** | Water, HU | 0.98 | 1.46 | -0.3 | 1.85 | 3.57 | 3.38 | 4.6 |
|  | Polystyrene, HU | –104.63 | –100.31 | –97 | –97.32 | –96.42 | –96.66 | –93.4 |
|  | Air, HU | –996.43 | –995.1 | –995.1 | –997.3 | –995.1 | –995 | –992.7 |
| **Pelvis Large** | Acrylic, HU | 121.33 | 121.3 | 121.4 | 128.2 | 121.3 | 121.4 | 128.6 |
| **140 kV** | Bone, HU | 917.38 | 912.4 | 911.9 | 911.8 | 912.3 | 911.9 | 901.3 |
| **mAS 1657.5** | Water, HU | 1.86 | 1.3 | 1.1 | -1 | 1.5 | 1.2 | 6.7 |
|  | Polystyrene, HU | –92.73 | –96.2 | –95.4 | –96.8 | –96.3 | –95.4 | –93 |

Abbreviations: HS-iCBCT, HyperSight iterative cone-beam CT reconstruction; MAR, metal artifact reduction; FDK, Feldkamp-Davis-Kress reconstruction; HU, Hounsfield units.
